# How Prepared Are We for Possible Bioterrorist Attacks: An Approach from Emergency Medicine Perspective

**DOI:** 10.1155/2018/7849863

**Published:** 2018-07-08

**Authors:** Ali Kemal Erenler, Murat Güzel, Ahmet Baydin

**Affiliations:** ^1^Hitit University, Department of Emergency Medicine, Çorum, Turkey; ^2^Samsun Education and Research Hospital, Department of Emergency Medicine, Samsun, Turkey; ^3^Samsun Ondokuzmayıs University, Department of Emergency Medicine, Samsun, Turkey

## Abstract

Preparedness for bioterrorist attacks and early recognition of specific agents are essential for public health. Emergency departments may play an important role in this field. The large spectrum of bioterrorism involves not only disastrous terrorism with mass casualties, but also microevents using low technology but producing civil unrest, disruption, disease, disabilities, and death. It aims not only to cause mortality and morbidity, but also to lead to social and political disruption. Preparedness appears to be the most potent defense against possible bioterrorist events. In this article, we aim to create awareness against biological agents and underline the importance of emergency departments in this public health problem.

## 1. Introduction

Bioterrorism is a term used for intentional use of pathogenic strains of microbes to cause disease or death in living things and/or to give harm to environment [1, 2]. These agents can typically be found in nature, but it is possible that they could be altered to increase their ability to cause disease, enhance their resistance to current medicines, or increase their ability to be spread into the environment [3]. Bioterrorism covers a large spectrum of concerns, from catastrophic terrorism with mass casualties to microevents using low technology but producing civil unrest, disruption, disease, disabilities, and death [4]. The aim of bioterrorism is not only to cause mortality and morbidity, but also to lead to social and political breakdown. Since it is a threat of the 21^st^ century, it is important to be aware of the biological features of the instruments of the war [5]. The most important defensive measure that can be taken seems to educate front line healthcare providers involving nurses, doctors, and clinicians. It is clear that there is a need for a rapid surveillance system between hospitals, emergency rooms, laboratories, and public health departments. As the first responders, a well-trained staff, efficient data systems, and sufficient laboratory capacity in the ED are the essentials for an appropriate response [6]. In this review, we aimed to emphasize the importance of early recognition of a bioterrorist attack and determine the measures that can be taken in emergency department.

## 2. History

Bioterrorism has a long history. To our knowledge, the potential impact of infectious diseases on people and armies has been recognized since 600 BC [7]. During the leaguer of Kaffa, Feodosiya, Ukraine, the Tartars (Mongols), who attacked Kaffain in the 14^th^ century, tossed dead and dying plague victims into the city in an attempt to spread the disease. Armies polluted the water by throwing dead animals into water supplies. The incident was reported by an Italian, Gabriele de' Mussi, who probably based his narrative on eyewitness accounts of survivors of the attack who fled Kaffa [8]. the plague pandemic also known as the “Black Death” which rapidly extended through Europe and North Africa initiated following migration of refugees from the defeated city [5]. In the World War I, the US and Germany tried to develop biological weapons to contaminate animal fodder [7]. British troops exposed native Indians with blankets and linens used by smallpox victims to deliberately spread smallpox among Indians in the Ohio River Valley [9]. In the Cold War, the US and the Soviet Union created arsenals of biological agents for use in battle and against people [7]. Between 1915 and 1916, Doctor Anton Dilger, on behalf of the German Government, worked with cultures of anthrax and glanders with the intention of biological sabotage [10]. In 1984, the pseudo-Buddhist Rajneeshee cult distributed Salmonella in restaurants and grocery stores in Oregon to poison civic leaders and seize control of the local Government. In 1992, Russia had the ability to launch missiles containing weapons-grade small pox. Some terrorist organizations, including Al-Qaeda, have explored the use of biological agents. In 1995, The Tokyo subway was attacked by Sarin gas, by the religious sect Aum Shinrikyo. With this attack, 12 people have died and 5000 people have been hospitalized. In 2001, letters containing anthrax spores were mailed to a television news anchor, US senator, and others. These letters led to the death of a few people and hospitalization of a few others [7]. It is also known that the cult was cultured and experimented with botulin toxin, anthrax, cholera, and Q fever. They have also sent healthcare providers to Africa on a mission; however, their main purpose was to bring back Ebola virus samples to use as a biological weapon [11].

Agents used for bioterrorism are summarized in Table 1.

## 3. Detection

After the attack in the US with Anthrax spores in the letters in 2001, the necessity of detection and decontamination of critical facilities appeared. During the recent decade, a remarkable progress in the detection, protection, and decontamination of biological warfare agents has emerged since various and sophisticated detection and decontamination methods have been developed and implemented [12]. In case of an attack, many people can be affected in a short period of time and great chaos may occur on the healthcare system [13]. In order to avoid the logistical problems and insufficiency of medications and resources, the US Centers for Disease Control (CDC) encourages healthcare professionals to be familiar with warfare agents, and in association with governmental organizations have implemented a “Bioterrorism Preparedness and Response Program” to detect and appropriately respond to a potential bioterrorist attack, immediately [14]. Early recognition is the main principle in minimizing casualties, initiating appropriate therapy, and preserving adequate resources. However, symptoms and signs following exposure to warfare agents are often nonspecific and can easily be mistaken for common diseases seen in the emergency departments every day [15]. There is a strong relationship between identifying a potential bioterrorism event and to maintain a strong index of suspicion [16]. Rapid and accurate technologies have to be developed and have to confirm the presence of these agents unambiguously in different ways. Identification of a biothreat agent in very low concentrations is essential. It also should have the possibility to be detected in various matrices. In addition, it should be portable, easy to use, and efficient to detect multiple threat agents. Any of the available systems are not capable of meeting all these criteria and methodology has to be chosen according to the situation. Development of detection systems that can determine the biological agents in potent concentrations is a challenge, and due to nonsensitive antigen and antibody based systems, research is focused on development of nucleic acid based sensors that are much more sensitive but need complex sample preparation [17]. Recently, there is a rising necessity to specify markers for specific agents that are appropriate for use in healthcare facilities and emergency departments.

## 4. Role of Emergency Department

A secure communication pathway in association with health departments and public health officials to outbreaks of bioterrorist events may be created between emergency services and governmental public health departments. Communication between medical health providers, emergency room personnel, infection-control personnel, and infectious-disease personnel in hospitals might be constituted and maintained through a health advisory network. In addition, at regular intervals, meetings may be performed in order to share information regarding planning responses in collaboration against bioterrorist attacks. Local health authorities, including emergency services, should examine their preparedness repeatedly for a potential bioterrorist attack and routinely review coordination issues with agencies that take place in response. Programs aimed to coordinate and direct emergency preparedness and response including antibioterrorism efforts should be created [18]. Training programs for health providers are also useful for preparedness and alertness. It was shown that when gaming simulations were used to test knowledge and skill of individuals who engage in antibioterrorism, better outcomes were obtained with trained personnel [19]. Association of better scores with training underline the importance of training programs when a real event is faced. However, in a survey study with 1028 participants in Canada, it was reported that most of the emergency service providers have not been trained enough to identify and work in contaminated environments under chemical, biological, radiological, and nuclear attacks [20]. The situation must be considered worse in developing countries, and programs focusing on health providers, especially those in emergency services, must be initiated as quickly as possible. Public health providers can be trained either by online education programs or face to face [21]. Surveillance systems can also be developed to provide important new capabilities in responding to public health emergencies. However, these efforts may result in false alarms and related increased cost [22].

Measures to enhance diagnostic and therapeutic capabilities and capacities alongside training and education are thought to improve the ability of society to combat 'regular' infectious diseases outbreaks, as well as mitigating the effects of bioterrorist attacks [23].

When a public health emergency is identified, it may be wise to redirect resources, e.g., funding, staff, and space from core public health programs to contribute to the public health emergency response [24].

There are also studies in the literature suggesting step by step measures to address bioterrorist events. Current trends in biosecurity and cybersecurity include (1) the wide availability of technology and specialized knowledge that previously were available only to governments; (2) the global economic recession, which may increase the spread of radical non-state actors; and (3) recent US and EU commission reports that reflect concerns about non-state actors in asymmetric threats. The nature of bioterrorism threats requires collaboration across several sectors including intelligence, police, forensics, customs, and other law enforcement organizations who must work together with public and animal health organizations as well as environmental and social science organizations. Coordination in decision-making among these organizations is required, based on knowledge and information sharing. An “information sharing risk-benefit analysis” may be constituted to determine the risk of not sharing information among organizations compared to the benefit of sharing information in order to prevent a terrorist attack and to enhance a rapid response capability in case it occurs. In work package 3 of the EU project AniBioThreat, early warning is the main topic. A strategy has been generated based on an iterative approach to bring law enforcement agencies and human and animal health institutes together. Workshops and exercises were involved during the first half of the project, and spin-off activities include new preparedness plans for institutes and the formation of a legal adviser network for decision making. Additionally, in Stockholm, Sweden, in 2012, a seminar on actionable knowledge was held, which identified the need to bring various agency cultures together to work on developing a resilient capability to identify early signs of bio- and agroterrorism threats. The seminar concluded that there are a number of challenges in building a collaborative culture, including developing an education program that supports collaboration and shared situational awareness [25].

Policy makers must also focus on vaccine production since vaccines are the best protection against infectious diseases. There is an ongoing academic debate on use of vaccines in biological warfare. Uncertainty of the threats is the major challenging issue in vaccine development. Even though vaccines against smallpox, anthrax, and Ebola viruses seem to have priority, an extensive policy on vaccines covering both military personnel and civilians is needed [6].

## 5. Conclusion

Preparedness appears to be the most potent defense against possible bioterrorist events [5].

Reports reveal that we are not well-prepared to deal with a terrorist attack that employs biological weapons. As was done in response to the nuclear threat, the medical community should educate the public and policy makers about the threat. In the longer term, we need to be prepared to detect, diagnose, characterize epidemiologically, and respond appropriately to biological weapons use and the threat of new and reemerging infections. On the immediate horizon, we cannot delay the development and implementation of strategic plans for coping with civilian bioterrorism. [26]. As a result, education and training of the healthcare providers, especially emergency physicians, are the mainstay of the battle against bioterrorism. Emergency departments must be constructed in a way suitable for a possible chaos and overcrowding that may occur when a real event happens. Awareness and preparedness to biological warfare agents must be accepted as a part of national biodefense policy.

## Figures and Tables

**Table 1:** Classification of diseases and agents according to CDC guidelines.

**Categories**	**Diseases/Agents**
**A**	Anthrax (*Bacillus anthracis*), Botulism (*Clostridium botulinum*), Plague (*Yersinia pestis*), Smallpox (*Variola major*), Tularemia (*Francisella tularensis*), Viral hemorrhagic fevers

**B**	Brucellosis (*Brucella* species), epsilon toxin of *Clostridium perfringens*, food safety threats (e.g., *Salmonella* species, *Escherichia coli* O157:H7, Shigella), Glanders (*Burkholderia* mallei), melioidosis (*Burkholderia pseudomallei*), Psittacosis (*Chlamydia psittaci*), Q fever (*Coxiella burnetii*), ricin toxin from *Ricinus communis* (castor beans),Staphylococcal enterotoxin B, Typhus fever (*Rickettsia prowazekii*), viral encephalitis [alphaviruses (e.g. Venezuelan equine encephalitis, eastern equine encephalitis, western equine encephalitis)], and water safety threats (e.g. *Vibrio cholerae*, *Cryptosporidium parvum*)

**C**	*Nipah virus*,* Hantavirus*
